# Challenges encountered using standard vector control measures for dengue in Boa Vista, Brazil

**DOI:** 10.2471/BLT.13.119081

**Published:** 2014-07-24

**Authors:** Rafael Maciel-de-Freitas, Denise Valle

**Affiliations:** a Laboratório de Transmissores de Hematozoários, Instituto Oswaldo Cruz-Fiocruz, Fiocruz, Av. Brasil, 4365, Manguinhos, Rio de Janeiro, Brazil, CEP 21040-360.; b Laboratório de Biologia Molecular de Flavivirus, Instituto Oswaldo Cruz-Fiocruz, Rio de Janeiro, Brazil.

## Abstract

**Problem:**

In 2010, dengue virus (DENV) serotype–4 was detected during a dengue outbreak in the Amazonian city of Boa Vista. At that time Brazil was already endemic for DENV-1, DENV-2 and DENV-3. This was the first time DENV-4 was observed in the country after it was initially detected and eliminated in 1981.

**Approach:**

To hinder the spread of DENV-4 throughout Brazil, standard vector control measures were intensified. Vector control professionals visited 56 837 households in 22 out of 31 districts of Boa Vista, to eliminate mosquito-breeding sites. Water storage containers were treated with the larvicide diflubenzuron, and deltamethrin was sprayed for adult *Aedes aegypti* mosquitoes. Fifteen days later, a second larvae survey and additional deltamethrin applications were performed.

**Local setting:**

In Brazil, dengue vector control is managed at all three government levels. Regular surveillance of *Aedes aegypti* is done four to six times a year to strengthen mosquito control activities in areas with high-vector density. Educational dengue control campaigns in communities are scarce, especially between outbreaks.

**Relevant changes:**

In spite of extensive implementation of all standard control actions recommended by the Brazilian dengue control programme, only a slight decrease in mosquito density was detected.

**Lessons learnt:**

There is a need to redesign all levels of dengue control. Public consultation and engagement, behaviour change and actions that go beyond technical impositions are required. Vector control programme managers need to reflect on what constitutes good practices and whether intermittent information campaigns are effective measures for dengue prevention and control.

## Résumé

**Problème:**

En 2010, le sérotype-4 du virus de la dengue (DENV) a été détecté pendant une épidémie de dengue dans la ville amazonienne de Boa Vista. À l'époque, le Brésil était déjà endémique pour les sérotypes DENV-1, DENV-2 et DENV-3. C'était la première fois que le sérotype DENV-4 était observé dans le pays après avoir été initialement détecté et éliminé en 1981.

**Approche:**

Pour empêcher la propagation de DENV-4 dans tout le Brésil, les mesures standard de lutte antivectorielle ont été intensifiées. Des professionnels de la lutte antivectorielle ont rendu visite à 56 837 ménages dans 22 des 31 districts de Boa Vista pour éliminer les sites de reproduction des moustiques. Les réservoirs d'eau ont été traités avec le larvicide diflubenzuron, et de la deltaméthrine a été pulvérisée pour éliminer les moustiques adultes *Aedes aegypti*. Quinze jours plus tard, une deuxième évaluation des larves et des applications supplémentaires de deltaméthrine ont été effectuées.

**Environnement local:**

Au Brésil, la lutte antivectorielle de la dengue est gérée aux trois niveaux gouvernementaux. La surveillance régulière des moustiques *Aedes aegypti* est effectuée 4 à 6 fois par an pour renforcer les activités de lutte contre les moustiques dans les zones à densité élevée de vecteurs. Les campagnes d'information pour lutter contre la dengue dans les communautés sont rares, en particulier entre les épidémies.

**Changements significatifs:**

Malgré la mise en œuvre étendue de toutes les actions standard de lutte recommandées par le programme brésilien de lutte contre la dengue, seule une légère diminution de la densité des moustiques a été détectée.

**Leçons tirées:**

Il est nécessaire de redessiner tous les niveaux de la lutte contre la dengue. La consultation et l'engagement du public, le changement de comportement et des actions qui vont au-delà des impositions techniques sont requis. Les directeurs des programmes de lutte antivectorielle doivent réfléchir sur ce qui constitue de bonnes pratiques et évaluer si les campagnes intermittentes d'information sont des mesures efficaces pour la prévention et la lutte contre la dengue.

## Resumen

**Situación:**

En el año 2010 se detectó el serotipo 4 del virus del dengue (DENV) durante un brote de dengue en la ciudad amazónica de Boa Vista. En aquel momento, en Brasil ya eran endémicos los serotipos DENV-1, DENV-2 y DENV-3. Tras su detección inicial y eliminación en 1981, esta fue la primera vez que se volvía a observar el DENV-4 en el país.

**Enfoque:**

Con objeto de impedir la propagación del DENV-4 por todo Brasil, se intensificaron las medidas de control vectorial estándar. Profesionales del control vectorial visitaron 56 837 hogares en 22 de los 31 distritos de Boa Vista para eliminar los criaderos de mosquitos. Los recipientes de almacenamiento de agua se trataron con el larvicida diflubenzuron, y se roció deltametrina contra los mosquitos adultos *Aedes aegypti*. Quince días más tarde se llevó a cabo una segunda encuesta de larvas y se realizaron aplicaciones adicionales de deltametrina.

**Marco regional:**

En Brasil, el control vectorial del dengue se gestiona en los tres niveles gubernamentales. El control regular de *Aedes aegypti* se realiza de cuatro a seis veces al año para reforzar las actividades de control de los mosquitos en áreas con una densidad vectorial elevada. Las campañas educativas en las comunidades sobre el control del dengue son escasas, especialmente entre los brotes.

**Cambios importantes:**

A pesar de la amplia aplicación de todas las acciones de control estándar recomendadas por el programa de control del dengue brasileño, únicamente se detectó una disminución leve en la densidad de mosquitos.

**Lecciones aprendidas:**

Hay una necesidad de rediseñar todos los niveles de control del dengue. Son necesarios una consulta y compromiso públicos, un cambio en el comportamiento y acciones que vayan más allá de las imposiciones técnicas. Los directores de los programas de control vectorial deben reflexionar sobre qué constituye las buenas prácticas y si las campañas de información intermitentes son medidas eficaces para controlar y prevenir el dengue.

## ملخص

**المشكلة:**

في عام 2010، تم اكتشاف النمط المصلي 4 من فيروس حمى الضنك (DENV) خلال فاشية لحمى الضنك في مدينة بوا فيستا الواقعة على نهر الأمازون. وفي هذا الوقت، كانت الأنماط المصلية 1 و2 و3 من فيروس حمى الضنك متوطنة بالفعل. وكانت هذه أول مرة يلاحظ فيها النمط المصلي 4 من هذا الفيروس في البلد بعدما تم اكتشافه في البداية واستئصاله في عام 1981.

**الأسلوب:**

تم تكثيف التدابير الموحدة لمكافحة النواقل لإعاقة انتشار النمط المصلي 4 من فيروس حمى الضنك في جميع أرجاء البرازيل. وقام العاملون في مكافحة النواقل بزيارة 56837أسرة في22 من أصل 31 منطقة في بوا فيستا، لاستئصال مواقع تكاثر البعوض. وتم معالجة حاويات تخزين المياه باستخدام الدايفلوبنزرون المبيد لليرقات وتم رش دلتاميثرين على بعوضة الزاعجة المصرية البالغة، وأجري بعدها بخمسة عشر يوماً مسحاً ثانياً عن اليرقات واستعمالات الدلتاميثرين الإضافية.

**المواقع المحلية:**

يتم تنفيذ التدبير العلاجي لمكافحة نواقل حمى الضنك على جميع المستويات الحكومية الثلاثة. ويجرى الترصد المنتظم لجنس البعوضة الزاعجة المصرية من أربع إلى ست مرات سنوياً لتعزيز أنشطة مكافحة البعوض في المناطق ذات الكثافة العالية من النواقل. وتندر الحملات التثقيفية لمكافحة حمى الضنك في المجتمعات المحلية، لا سيما بين الفاشيات.

**التغيّرات ذات الصلة:**

على الرغم من التنفيذ الشامل لجميع إجراءات المكافحة الموحدة التي أوصى بها برنامج مكافحة حمى الضنك في البرازيل، كان الانخفاض المكتشف في كثافة البعوض طفيفاً.

**الدروس المستفادة:**

ثمة حاجة لإعادة تصميم جميع مستويات مكافحة حمى الضنك. ويتعين وجود مشاورات ومشاركة عامة وتغيير للسلوك والإجراءات تتخطى العوائق التقنية. ويجب على مدراء برنامج مكافحة النواقل التفكير ملياً للتعرف على ما يشكل الممارسات الجيدة وما إذا كانت حملات التثقيف المتقطعة تمثل تدابير فعالة لتوقي حمى الضنك ومكافحتها.

## 摘要

**问题:**

2010年在亚马逊河城市博阿维斯塔爆发登革热期间检测到登革热病毒（DENV）血清分型-4。当时，巴西已经发生了DENV-1、DENV-2和DENV-3地方病。这是继1981年最初检测并消灭DENV-4之后，首次在该国发现DENV-4。

**方法:**

为防止DENV-4在巴西的全面蔓延，加强了标准病媒控制措施。病媒控制专业人员走访了博阿维斯塔31个区中22个区的56 837户家庭，以消除蚊虫孳生地点。使用杀幼虫剂二氟脲处理储水容器，喷洒溴氰菊酯对付成年埃及伊蚊。十五天后，执行第二次幼虫调查，并施用额外的溴氰菊酯。

**当地状况:**

巴西在所有三个政府层面进行登革热病媒控制的管理。每年进行四到六次埃及伊蚊定期监测，以便加强病媒密度高地区的蚊虫活动控制。社区中教育性的登革热防治活动非常少，在疫情爆发之间尤其如此。

**相关变化:**

尽管广泛实施巴西登革热防治计划建议的所有标准控制行动，仅检测到蚊虫密度的轻微降低。

**经验教训:**

需要重新设计登革热控制的各个层面。需要有公共协商和参与、行为改变和超越技术实施的行动。病媒控制项目管理人员需要反思什么才是良好实践的构成要素，间断性的信息宣传活动是否是登革热预防和控制的有效措施。

## Резюме

**Проблема:**

В 2010 году, во время вспышки лихорадки денге в городе Боа-Виста в бассейне реки Амазонки, был обнаружен вирус денге 4-го серотипа (DENV-4). К этому времени Бразилия уже являлась эндемичной по серотипам DENV-1, DENV-2 и DENV-3. Серотип DENV-4 наблюдалсяв страневпервыепосле первоначального выявления ​​и ликвидации в 1981 году.

**Подход:**

Для воспрепятствования распространению серотипа DENV-4 по всей Бразилии, были усилены стандартные меры по борьбе с переносчиками инфекций. Для уничтожения мест размножения комаров специалисты службы по борьбе с переносчиками инфекций посетили 56 837 домохозяйств в 22 из 31 района города Боа-Виста. Контейнеры для хранения воды обрабатывались ларвицидом дифлубензурон, а для борьбы со взрослыми особями желтолихорадочных комаров*AedesAegypti* распылялся дельтаметрин. Спустя 15 дней было проведено второе исследование на предмет наличия личинок и произведена дополнительная обработка дельтаметрином.

**Местные условия:**

В Бразилии борьба с переносчиками лихорадки денге контролируется на всех трех правительственных уровнях. Надзор за *AedesAegypti* проводится регулярно 4-6 раз в год для усиления мероприятий по борьбе с комарами в районах с высокой плотностью переносчиков инфекций. Просветительские мероприятия по контролю залихорадкой денге в сообществах проводятся редко, особенно в периоды между вспышками.

**Осуществленные перемены:**

Несмотря на широкомасштабное проведение всех стандартных мероприятий по борьбе с переносчиками инфекций, рекомендованных Бразильской программой по борьбе с лихорадкой денге, удалось достичь лишь незначительного снижения плотности комаров.

**Выводы:**

Необходим пересмотр мероприятий по борьбе с лихорадкой денге на всех уровнях. Требуется участие и проведение консультаций с общественностью, изменение модели поведения и действия, выходящие за рамки технических мер. Руководителям программы по борьбе с переносчиками инфекций необходимо проанализировать передовые практики и решить, являются ли периодические информационные кампании эффективной мерой по профилактике и борьбе с лихорадкой денге.

## Introduction

Dengue is currently the arbovirus affecting the highest number of people worldwide. The World Health Organization (WHO) estimates that 50–100 million dengue infections occur annually, and that about 2.5 billion people are at risk of infection.^1^ Currently, there are four distinct serotypes of the dengue virus (DENV) that infect humans.^1^ Infection with one dengue serotype induces permanent immunity against that serotype, but not against the others. A person infected with the virus for the first time can develop high fever together with rash or headache and eye, joint, muscle or bone pain. However, a sequential infection with another serotype increases the risk of developing severe dengue, with potentially deadly complications.

In 1981, a dengue outbreak caused by DENV-1 and DENV-4 occurred in Boa Vista in northern Brazil. Local vector control measures successfully contained the virus, probably because of the geographic isolation of Boa Vista at that time. However, since the introduction of DENV-1 into the State of Rio de Janeiro in 1986, dengue has become a nationwide public health problem, with more than 60% of all Latin American cases of dengue occurring in Brazil.^2^ Since then, DENV-2 and DENV-3 have also been detected in Rio de Janeiro State, highlighting this area as the port of entry and dissemination of DENV in Brazil.^3^

**Fig. 1:**
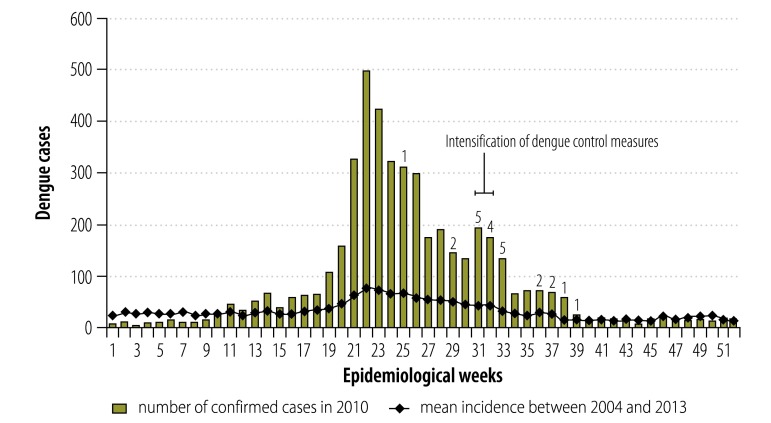
Dengue in the city of Boa Vista, Brazil, 2004–2013

Notes: The numbers above the bars represent persons reporting the first symptoms of dengue that were later confirmed as dengue-4. The first dengue-4 case was confirmed at week 31.

In a 2010 dengue outbreak in Boa Vista, DENV-1 and DENV-2 were co-circulating. At the end of the outbreak, DENV-4 was detected in the serum sample from a patient who had presented clinical symptoms seven weeks before (Fig. 1). This was the first time DENV-4 was observed in Brazil after it was initially detected in Boa Vista in 1981.^4^^,^^5^

## Approach

Immediately after the confirmation of DENV-4, intensification of regular vector control actions were started in Boa Vista to reduce the density of *Aedes aegypti* and hinder dissemination of this serotype in the country. Standard vector control protocols, recommended by the Brazilian dengue control programme, were followed.^6^ Source reduction was performed in all houses in 22 out of 31 districts of Boa Vista. These districts, which covered 75% of all habitations, included all those where DENV-4 cases had been diagnosed or there had been a history of repeated dengue outbreaks. After householders had given oral consent, all potential breeding sites identified in each dwelling were inspected for larvae by vector control professionals. Whenever possible, potential breeding sites were removed, as recommended by WHO.^7^ Permanent water containers (e.g. tanks, wells and pools) were treated with diflubenzuron.^8^ Larvae samples were brought to the laboratory to identify the species. A second larval survey was conducted 15 days later in 10% of the habitations of the same 22 districts. As in the first survey, these actions were accompanied by vehicles mounted with an ultra-low volume sprayer to administer 2% deltamethrin against adult mosquitoes.^9^

## Local setting

All three government levels (federal, state and municipality) share responsibility for dengue control in Brazil. The federal level provides guidelines for vector control, allocates resources to the states and purchases insecticides and equipment, such as vehicles mounted with an ultra-low volume sprayer to support chemical control. The states assist and supervise municipalities, acquire consumables and small equipment, such as nylon nets or lids for water tanks or mosquito traps, and gather information about the municipalities to notify the Health Ministry. The municipality is responsible for operations such as management of vector control professionals and actions, following central level recommendations. In practice, this shared responsibility can reduce the efficiency of vector control; for example, decision-making processes can be bureaucratic and time-consuming.

In Brazil, routine surveillance of *Ae. aegypti* is based on the larval index rapid *Aedes* assay. This assay consists of random sampling of a proportion of dwellings (3.5–4%) per district over a period of up to one week and is performed to get a snapshot of the infestation scenario. The surveillance is carried out four to six times each year in the municipalities most affected by dengue. Larval registers are used as an *Ae. aegypti* infestation indicator.^10^ These registers take into account both house index (i.e. number of positive houses per total of inspected houses) and Breteau index (i.e. number of breeding sites per total of inspected houses). Mosquito control activities are then strengthened in the areas with higher mosquito density.

In Brazil, ultra-low volume space spraying is recommended only during dengue outbreaks.^7^ However, mosquito adulticides are used as the primary vector-control tool in some municipalities, resulting in high pyrethroid resistance rates,^11^ leading to decreased chemical control effects during outbreaks when the adulticides are most needed.

During periods between epidemics, few resources are allocated to increase awareness in the affected communities regarding the importance of dengue prevention and almost no regular educational efforts are conducted. One exception was the “D-Day Against Dengue”, a mobilization initiative originally performed once a year, just before the dengue season. This campaign was abandoned because it encouraged people to have good dengue practices on one day each year, rather than continually.

## Relevant changes

Confirmation of DENV-4 in Boa Vista led to the rapid formation of a committee with municipal, state and federal health secretaries, which ensured that all standard control actions recommended by the Brazilian dengue control programme were accomplished.

Intensification of vector control in Boa Vista included inspection of 56 837 houses, 10% of them were visited again 15 days later. 94 325 containers were removed from these houses or treated with diflubenzuron. Most positive containers (601/1017) were classified as miscellaneous receptacles (usually domestic garbage items that could become small isolated breeding sites).^9^ Concomitantly, ultra-low volume deltamethrin spraying was done in the affected areas. However, only a slight decrease in vector density was detected; the house index was reduced from 1.7 before interventions to 1.37 immediately after the second survey.^9^ This reduction did not result in a significant change in the seasonal dynamics of dengue, when taking into account the history of cases in the municipality (Fig. 1). This could be due to highly-productive cryptic containers that were not inspected by vector control professionals. Additionally, the rapid twofold to threefold increase in the resistance rate of the local *Ae. aegypti* adults indicates a strong selection of resistant mosquito populations due to intense insecticide application.^9^ These results highlight the low efficacy of standard recommended vector control measures.

## Lessons learnt

In Boa Vista, simply intensifying routine vector control measures to stop DENV-4 spreading throughout Brazil was unsuccessful. The low efficacy of these measures in reducing *Ae. aegypti* density points to the need to change the control programme at all levels (Box 1). There are no simple solutions, but it is expected that the basis of effective actions for prevention and control will be changes in behaviours and attitudes of both dengue control managers and the affected population, with a strong commitment to eliminating breeding sites and avoiding contact with mosquitoes. An example of more effective dengue control measures comes from Singapore, where in 2004–2005, a massive community initiative was carried out by volunteers from government agencies and nongovernmental organizations to eliminate breeding habitats. This initiative was linked to a strong interagency dengue task force and is a well-known example of a powerful reduction of infestation levels and, consequently, of a highly-significant decrease in the incidence of dengue outbreaks.^12^

Box 1 **Summary of main lessons learnt**

- A proactive attitude from both public health managers and the general population towards the correct identification and elimination of vector breeding sites is still the cornerstone of dengue prevention.
- In *Ae. aegypti* control, the complementary nature of chemical insecticides and new technologies against adult mosquitoes must be kept in perspective to avoid the loss of effectiveness of such tools.
- Attempts to block outbreaks using insecticides in locations where resistance has been detected previously will rapidly increase and disseminate resistant vectors, hampering the effectiveness of insecticides for a long time.

The challenge in rapid, efficient and large-scale control of *Ae. aegypti* is to change current strategies and what are seen as good practices. For instance, where insecticide resistance is high at baseline, insecticide use during an outbreak should be reconsidered. Also, assuming that insecticides will be the primary means of vector control delivers a false sense of security to the local community and confuses overall vector control with chemical vector control. The use of insecticides must be considered as just one of the available measures for vector control and not the primary strategy to keep vector density low. Currently, new approaches to reducing dengue transmission are being tested, such as an endosymbiotic bacterium or transgenic mosquitoes.^13^^,^^14^ Although development of such strategies must be encouraged, it should not be forgotten that these complement good sanitation and health behaviour practices.

Brazil’s current dengue vector control programme is designed and run by the government and individuals are not usually encouraged to take responsibility for appropriate and continuous dengue control in their own dwellings. Given the *Ae. aegypti* life-cycle, inspection of all houses in all metropolitan regions is not feasible and vertically structured programmes have proved to have a low chance of success.^15^

Using current standard measures to urgently control for infestation during a dengue outbreak has been shown not to work. Effective control throughout the year will be achieved only if dengue control moves towards a more participative focus. Starting community-based control programmes in the low-transmission season may also help to keep mosquito density below a critical threshold during periods of high transmission.

In Boa Vista, as in several other municipalities, most receptacles containing larvae are small containers (i.e. domestic garbage cans). Therefore, community engagement efforts that focus on waste reduction, allied with appropriate removal of garbage, would decrease the availability of these breeding sites and, potentially, *Ae. aegypti* density.

Local input is needed to identify the best way to raise awareness and empower residents to accomplish effective and sustainable dengue prevention. Where a local community joins dengue-prevention activities, highlighting practical actions in their own dwellings and neighbourhoods, vector control efforts will improve. Effective dengue control requires public engagement with committed vector control professionals.
